# Tissue-Specific and Minor Inter-Individual Variation in Imprinting of *IGF2R* Is a Common Feature of *Bos taurus* Concepti and Not Correlated with Fetal Weight

**DOI:** 10.1371/journal.pone.0059564

**Published:** 2013-04-08

**Authors:** Daniela Bebbere, Stefan Bauersachs, Rainer W. Fürst, Horst-Dieter Reichenbach, Myriam Reichenbach, Ivica Medugorac, Susanne E. Ulbrich, Eckhard Wolf, Sergio Ledda, Stefan Hiendleder

**Affiliations:** 1 Chair for Molecular Animal Breeding and Biotechnology, Gene Center, Ludwig-Maximilians-University, Munich, Germany; 2 Department of Veterinary Medicine, University of Sassari, Sassari, Italy; 3 Laboratory for Functional Genome Analysis (LAFUGA), Gene Center, Ludwig-Maximilians-University, Munich, Germany; 4 Physiology Weihenstephan, Technische Universität München, Freising, Germany; 5 Bavarian State Research Center for Agriculture, Institute of Animal Breeding, Grub, Germany; 6 Chair of Animal Genetics and Husbandry, Faculty of Veterinary Medicine, Ludwig-Maximilians-University, Munich, Germany; 7 JS Davies Non-Mendelian Genetics Group, School of Animal and Veterinary Sciences, The University of Adelaide, Roseworthy Campus, Roseworthy, Australia; 8 Research Centre for Reproductive Health, Robinson Institute, The University of Adelaide, Roseworthy Campus, Roseworthy, Australia; CNRS, France

## Abstract

The insulin-like growth factor 2 receptor (IGF2R) is essential for prenatal growth regulation and shows gene dosage effects on fetal weight that can be affected by *in-vitro* embryo culture. Imprinted maternal expression of murine *Igf2r* is well documented for all fetal tissues excluding brain, but polymorphic imprinting and biallelic expression were reported for *IGF2R* in human. These differences have been attributed to evolutionary changes correlated with specific reproductive strategies. However, data from species suitable for testing this hypothesis are lacking. The domestic cow (*Bos taurus)* carries a single conceptus with a similar gestation length as human. We identified 12 heterozygous concepti informative for imprinting studies among 68 *Bos taurus* fetuses at Day 80 of gestation (28% term) and found predominantly maternal *IGF2R* expression in all fetal tissues but brain, which escapes imprinting. Inter-individual variation in allelic expression bias, i.e. expression of the repressed paternal allele relative to the maternal allele, ranged from 4.6−8.9% in heart, 4.3−10.2% in kidney, 6.1−11.2% in liver, 4.6−15.8% in lung and 3.2−12.2% in skeletal muscle. Allelic bias for mesodermal tissues (heart, skeletal muscle) differed significantly (*P*<0.05) from endodermal tissues (liver, lung). The placenta showed partial imprinting with allelic bias of 22.9−34.7% and differed significantly (*P*<0.001) from all other tissues. Four informative fetuses were generated by *in-vitro* fertilization (IVF) with embryo culture and two individuals displayed fetal overgrowth. However, there was no evidence for changes in imprinting or DNA methylation after IVF, or correlations between allelic bias and fetal weight. In conclusion, imprinting of *Bos taurus IGF2R* is similar to mouse except in placenta, which could indicate an effect of reproductive strategy. Common minor inter-individual variation in allelic bias and absence of imprinting abnormalities in IVF fetuses suggest changes in *IGF2R* expression in overgrown fetuses could be modulated through other mechanisms than changes in imprinting.

## Introduction

The multifunctional mannose 6 phosphate/insulin-like growth factor 2 receptor (M6P/IGF2R), hereafter referred to as IGF2R, mediates endocytosis and subsequent clearance or activation of a variety of ligands involved in the regulation of cell growth and motility, including insulin-like growth factor 2 and transforming growth factor *β* [1], [2].

The IGF2R gene shows developmental stage specific expression levels which are highest in the fetus and decline rapidly after birth [3]. Murine *Igf2r* is imprinted [4] and switches from biallelic to maternal expression during implantation [5], [6]. By the fetal stage, expression from the maternal allele is established in all tissues of the conceptus with the exception of brain, which escapes imprinting [7]–[9]. In contrast, imprinting of *IGF2R* in human remains controversial, with exclusive biallelic expression [10]–[14], maternal or biallelic expression [15]–[17] and partial imprinting [15], [16] reported for fetal and/or placental samples.

The fundamental role of *Igf2r* in prenatal growth regulation [18]–[20] suggests that quantitative variation in imprinting could affect phenotype via gene dosage effects [21]. Indirect evidence for such an effect was obtained in the sheep model where fetal overgrowth induced by embryo culture was associated with hypomethylation at a CpG site in an intronic sequence element (differentially methylated region 2, DMR2) implicated in *IGF2R* imprinting and down regulated *IGF2R* expression [22]. However, the imprinting status of *IGF2R* was not determined in this study [22].

Species differences in imprinting, in particular in the placenta, appear to be linked to differences in reproductive strategies, e.g. litter size, gestational length, maturity of newborns and lifetime reproductive capability [23]. The inconsistent data on *IGF2R* imprinting in human have been interpreted as evidence for a polymorphic trait [15], [17], where the observed minority of imprinted or partially imprinted specimens could signal an evolutionary transition to biallelic expression in the population [17]. It was further hypothesized that differences in imprinting of *Igf2r/IGF2R* between mouse and human could be a consequence of different reproductive strategies, including competition between multiple fetuses and the shorter gestation period requiring a more efficient placenta in mouse [17], [23]. However, comparative data from other species suitable for testing this hypothesis are lacking.

The domestic cow (*Bos taurus*) has a similar gestation length as human (∼280 days), carries a single conceptus with comparable maturity at birth, has a similar lifetime reproductive capability, and shows a conserved *IGF2R* gene structure with high sequence homology to human [24]. These features make the cow an excellent model for comparisons with human and mouse, but current *Bos taurus IGF2R* imprinting data is limited to two fetal liver samples of unspecified age [12]. In the present study, we determined the tissue-specific imprinting status of *IGF2R* in first trimester *Bos taurus* concepti generated *in-vivo* or *in-vitro.* We quantified variation in allele-specific expression bias, i.e. expression of the repressed paternal allele relative to the predominantly expressed maternal allele, determined methylation levels in DMR2, and analyzed relationships between expression bias and fetal phenotype in fetuses with or without overgrowth after *in-vitro* fertilization (IVF) procedures with embryo culture. Our data show that in all fetal tissues but brain, *Bos taurus IGF2R* is imprinted and predominantly expressed from the maternal allele as in mouse. However, in contrast to mouse, we detected partial imprinting in placenta that could be related to differences in reproductive strategy between cow and mouse [23]. Contrary to expectations, imprinting and DNA methylation were not affected by IVF with *in-vitro* embryo culture and there was no correlation between minor variation in inter-individual allele-specific expression bias and fetal weight.

## Results and Discussion

### Imprinting of *IGF2R* in Fetal Tissues and Placenta

**Figure 1:**
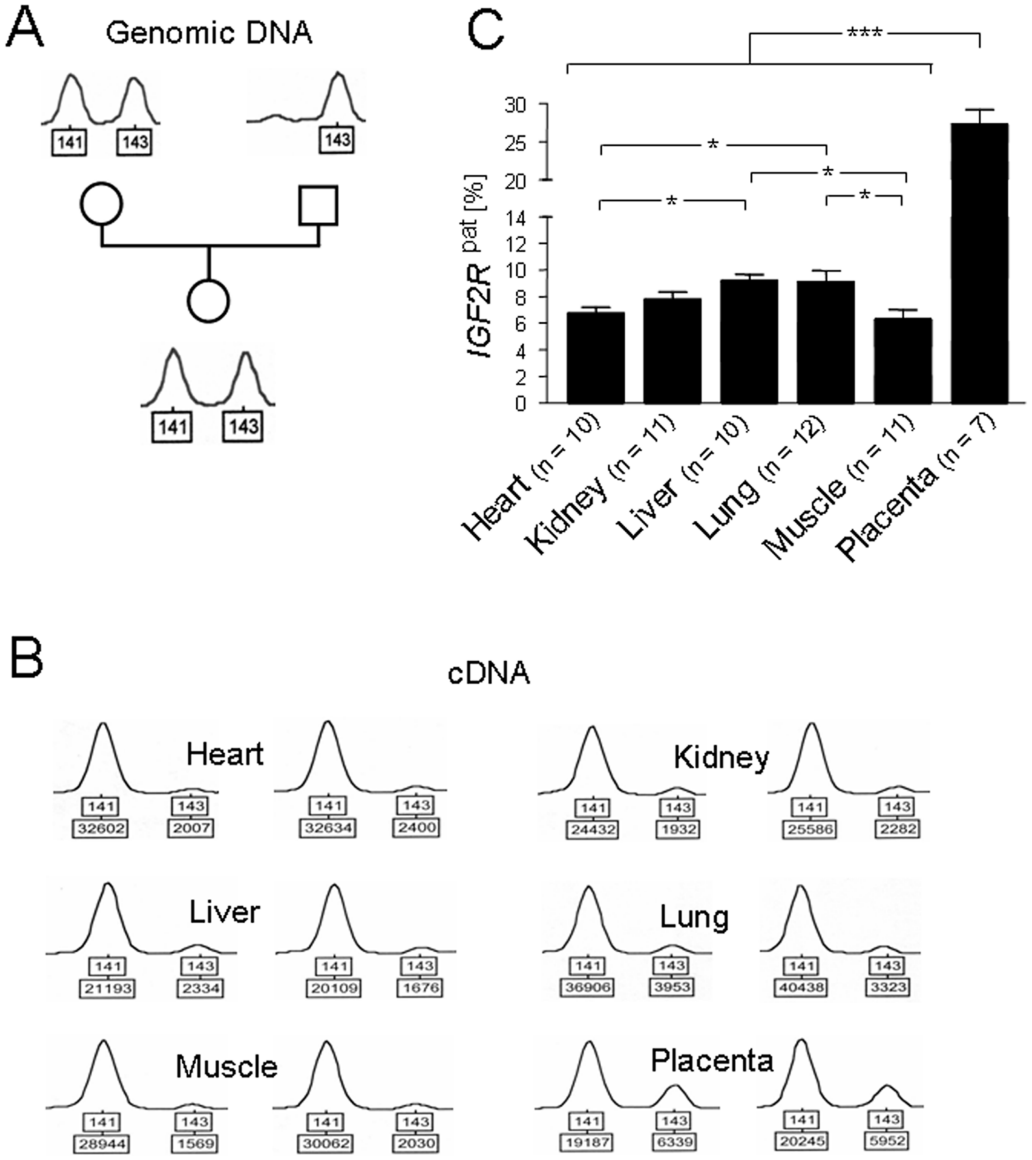
A–C: Imprinting of *IGF2R* in tissues of *Bos taurus* concepti recovered on Day 80 post fertilization. (**A**) Example of informative fetal and parental *IGF2R* microsatellite genotypes that allowed unequivocal identification of parental origin of alleles (boxed). (**B**) Examples of allelic expression levels (boxed, allele size at the top) determined for tissues from two heterozygous concepti. (**C**) Mean *IGF2R* allelic expression bias, i.e. expression of the paternal allele relative to the predominantly expressed maternal allele, in different tissues. Analysis of variance showed a significant tissue effect (*F*-test *P*<0.001) on allelic expression bias and least square means with standard errors of least square means were calculated for each tissue. Significant differences (*t*-tests) between tissues that originate from different cell lineages are indicated. *: *P*<0.05, ***: *P*<0.001.

Sequencing of bovine *IGF2R* fragments orthologous to human *IGF2R* exon 48 revealed a conserved microsatellite with the *Bos taurus* alleles (TG)_9/10_ (**Figure S1**). Genotyping of microsatellite alleles in 68 fetuses recovered on Day 80 post insemination (28% term) and of parental samples identified 12 heterozygous concepti with unequivocal maternal and paternal alleles (**Figure 1A**). Quantitative microsatellite analyses showed that *IGF2R* is not imprinted in bovine fetal brain but predominantly expressed from the maternal allele in all other fetal tissues and the placenta. Minor inter-individual variation in allele-specific expression bias, i.e. expression level of the repressed paternal allele, was observed in all tissues. Measured relative to the maternal allele, observed paternal expression ranged from 4.6−8.9% in heart, 4.3−10.2% in kidney, 6.1−11.2% in liver, 4.6−15.8% in lung, 3.2−12.2% in muscle and 22.9−34.7% in placenta (**Figure 1B, C**).

Low levels of paternal *Igf2r* expression were previously detected in transgenic and crossbred mouse fetuses [5], [6], but could be criticized on grounds of potential *Igf2r* transgene [5] and subspecies cross effects [25]. Measurements of paternal *IGF2R* expression in bovine fetal tissues clearly confirm the 5–10% of paternal expression estimated in mouse [5]. Furthermore, our data are consistent with the observation in mouse embryonic stem cells that establishment of imprinted gene expression increases transcription from the maternal allele up to 10-fold while transcription from the paternal allele remains constant [26].

**Figure 2:**
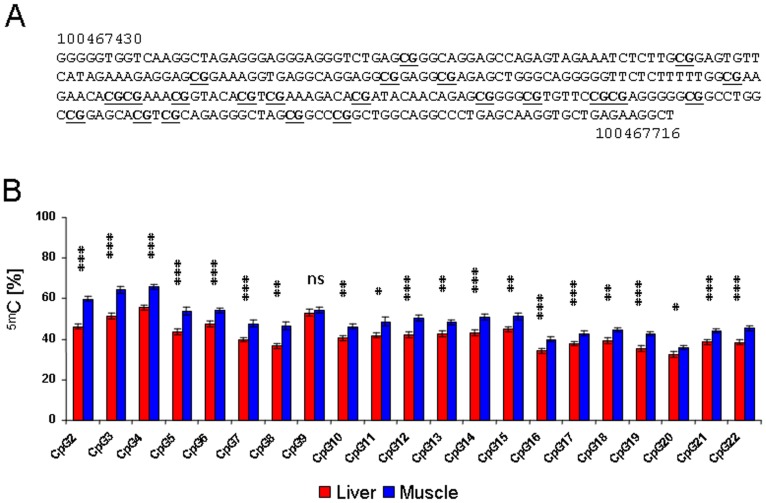
A,B: Analysis of *IGF2R* DMR2 DNA methylation levels by bisulfite pyrosequencing. (**A**) Sequence of PCR amplified 287 bp fragment of differentially methylated region 2 (DMR2) used in pyrosequencing. Nucleotide positions according to the NCBI *Bos taurus* reference genome assembly are indicated, CpGs are underlined. (**B**) Means and standard errors of means for methylation levels at 21 CpG sites in liver (n = 12) and muscle (n = 12) samples. Methylation at CpG1 could not be determined as it was 5′ of the internal pyrosequencing primers (see materials and methods) Significant differences between tissues are indicated: *: *P*<0.05, **: *P*<0.01, ***: *P*<0.001, ns: not significant.

Interestingly, our quantitative analysis of *IGF2R* imprinting revealed a significant tissue-effect on allele-specific expression bias (ANOVA *P*<0.001). Pair wise comparisons of least square means showed significant differences (*t*-tests *P*<0.05) between tissues originating from different cell lineages (**Figure 1C**). Tissues derived from mesoderm (heart, skeletal muscle) showed higher relative expression from the paternal allele than tissues derived from endoderm (liver, lung). Bisulphite pyrosequencing of CpG dinucleotides in DMR2 (**Figure 2A**) revealed significant DNA methylation differences between mesodermal (skeletal muscle) and endodermal (liver) tissue at 20 out of 21 surveyed sites (Paired samples *t*-tests *P*<0.05–*P*<0.001) (**Figure 2B**). Together with the biallelic expression found in ectoderm derived brain, this cell lineage effect suggested that in cow, similar to mouse [5], [6] and most likely sheep [27], imprinted expression of *IGF2R* in the embryo proper is not established until gastrulation.

Expression from the paternal *IGF2R* allele was 3–4 fold higher in placenta as compared with fetal tissues originating from the embryo proper (*P*<0.001) and can be classified as partial imprinting (**Figure 1B, C**). Since placenta and embryo proper derive from the two earliest lineages of the embryo, trophectoderm and inner cell mass, respectively, establishment of a trophectoderm-specific imprinted allelic expression bias prior to establishment of imprinted expression in the embryo proper (see above) could explain these differences. Alternatively, the high proportion of expression from the paternal *IGF2R* allele could indicate a transition from imprinted maternal expression in the first trimester of gestation to biallelic expression in later developmental stages. Temporary imprinting with intermediate states was reported for mouse and human *Slc22a3/SLC22A3,* which is in the *Igf2r/IGF2R* imprinted cluster with some shared control elements and mechanisms [**8]**, **[17]**, **[28**]. Future analyses of samples from more advanced developmental stages should help clarify this aspect.

Unlike the hemochorial placenta of mouse and human, the synepitheliochorial placenta in bovine presents a strong barrier that prevents mixing of fetal and maternal bloods and tissue [29]. Recent concerns about contamination of fetal placenta with maternal tissue [28], [30] nevertheless prompted us to test our sampling procedure for fetal placenta (cotyledon) for potential maternal contamination. We compared high copy number mitochondrial DNA (mtDNA) haplotypes of fetal placenta and maternal tissue from pregnancies with discordant mtDNA haplotypes after embryo transfer [31] and found no evidence for contamination with maternal tissue (not shown). Future studies need to address the possibility that different cell types in bovine fetal placenta [32] could have a different imprinting status and present as partially imprinted when analyzed in one sample.

The imprinted allelic expression bias in bovine placenta is clearly reminiscent of the partial imprinting reported for some fetal and placental samples in human [15], [16]. It has been speculated that inconsistent data on imprinting of *IGF2R* in human might stem from difficulties in interpreting exon 48 microsatellite data due to potential PCR artifacts [33]. However, a more recent report of polymorphic *IGF2R* imprinting in human term placentae was based on SNP sequencing [17] and thus excluded microsatellite artifacts. Furthermore, in the same study *SLC22A2,* which is located in the *IGF2R* imprinted cluster, showed polymorphic imprinting concordant with *IGF2R* imprinting status [17]. In the cow fetuses of the present study, the paternal microsatellite allele was always the longer allele (**Figure 1A and B**). Since *Taq* DNA polymerase slippage in TG/CA microsatellites favors repeat contraction over expansion by a factor of >14 [34], the bulk of any artifacts, if present, would be shorter than the original allele. Accordingly, we were able to detect a contracted fragment peak, but only when loading very large amounts of sample unsuitable for allele quantification, and found no evidence for repeat expansion (**Figure S2**). Thus, the paternal allele cannot be confounded with PCR artifacts in our analysis of paternal contributions to *IGF2R* expression.

### Imprinting of *IGF2R* after *in vitro* Embryo Production

**Figure 3:**
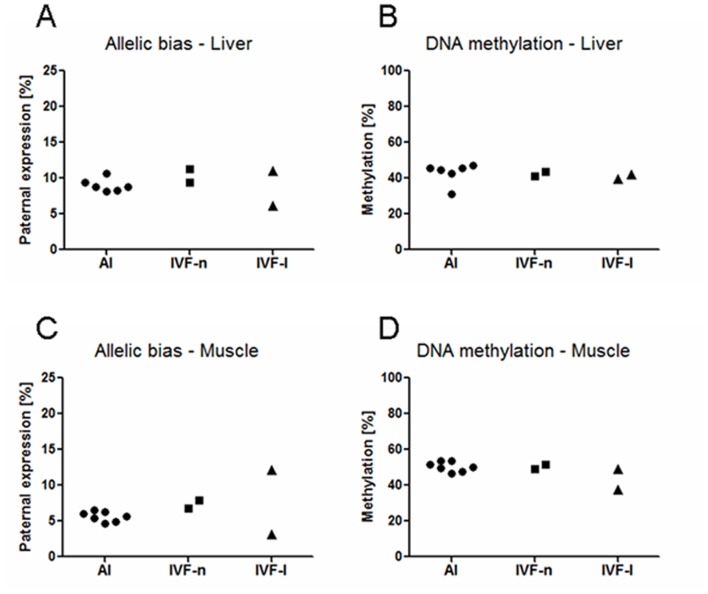
A–D: Examples of *IGF2R* allelic expression bias and DMR2 DNA methylation levels for samples from IVF fetuses and controls. Data points for in-vivo conceived control (AI) fetuses, normal weight in-vitro-fertilization (IVF-n) fetuses and overgrown in-vitro-fertilization (IVF-l) fetuses are shown. (**A,C**) Allelic expression bias for individual samples from liver and skeletal muscle. The Y axis represents expression from the paternal allele relative to the predominantly expressed maternal allele. (**B,D**) Corresponding DMR2 methylation levels measured in liver and skeletal muscle of the same individuals. Methylation levels are the mean for 21 CpG sites analyzed by bisulfite pyrosequencing (see **Figure 2**).

Four of the informative fetuses investigated in the present study were generated by *in-vitro* fertilization (IVF) procedures that involved embryo culture to the blastocyst stage [34]. Two of these IVF fetuses displayed fetal overgrowth (96.0 g and 105.8 g) and two showed normal weights (71.2 g and 89.8 g) based on comparisons with the eight fetuses conceived *in vivo* (mean 78.0 g, SD 4.49 g; heaviest fetus 86.8 g). Contrary to expectations from *IGF2R* expression and DMR2 DNA methylation data in overgrown sheep fetuses [22], which could be interpreted as evidence for changes in genomic imprinting [35], allelic expression ratios and DMR2 methylation levels of *IGF2R* in overgrown IVF, normal weight IVF and in-vivo conceived cow fetuses were similar (**Figure 3A–D**). Furthermore, we detected no significant correlations (*P*>0.10) between minor inter-individual variation in allelic expression bias and fetal weight. This suggested that changes in *IGF2R* expression in overgrown fetuses after in-vitro embryo culture [22] could be modulated through other mechanisms than changes in imprinting, perhaps through a canonical, transcription factor driven mechanism [21]. The first trimester fetuses used in the present study were in an early stage of fetal overgrowth and we cannot exclude the possibility that a more severe phenotype later in gestation could be correlated with measurable differences in imprinting. However, our findings are consistent with the limited available data on the role of imprinted genes in developmental plasticity which have so far generally failed to provide evidence for substantial relaxation of imprinting to explain altered expression of imprinted genes [21].

In conclusion, our data showed imprinted expression of *IGF2R* in cow fetuses with the exception of brain as in mouse. The partial imprinting observed in cow placenta could signal an effect of reproductive strategy as proposed by Monk *et al.* [17] and explored in detail by Frost and Moore [23] to explain differences in *IGF2R* imprinting between mouse and human. Tissue-specific quantitative variation in imprinting is a common feature of *IGF2R* expression in normal fetal development. The absence of imprinting abnormalities after IVF with embryo culture and lack of correlations between minor inter-individual variation in allelic bias and fetal weight suggest that changes in *IGF2R* expression in overgrown fetuses may be modulated through other mechanisms than changes in imprinting.

## Materials and Methods

### Fetal Tissue Samples

All experiments involving animals were performed in accordance with the relevant guidelines for the care and use of animals and with approval by the responsible animal welfare authority, the Regierung von Oberbayern. No animals were specifically generated for this study. Samples were obtained from fetuses generated by artificial insemination after estrus cycle synchronization and by in vitro fertilization procedures and embryo transfer as described previously [36]. Fetuses generated by *in-vitro* fertilization (IVF) with embryo culture were classified as overgrown when their weight exceeded the mean of control fetuses conceived *in-vivo* by more than four standard deviations. Brain samples were from the upper-left hemisphere of the telencephalon, liver samples from the *Lobus hepatis sinister*, and skeletal muscle samples from the *Musculus gluteus maximus*. Lung samples were obtained from the tip of the lungs and heart muscle was collected from the apex of the heart. The kidney sample consisted of one half of a bisected kidney. Cotyledon (*Placenta fetalis*) was sampled from placentomes close to the fetus. All samples were collected on ice and stored in RNAlater (Ambion, Austin, USA) at −80°C after 24 h incubation at 4°C.

### Detection of Microsatellite Polymorphism and Genotyping

Genomic DNA was extracted from parental blood or semen by standard protocols and from fetal liver tissue with the E.Z.N.A. Tissue DNA Mini Kit II (PEQLAB Biotechnologie GmbH, Erlangen, Germany). PCR was performed on 50–100 ng of DNA with 0.4 µM of each specific oligonucleotide primer (5′-6-FAM-CAGCTGGTGAAGTCAAAC-3′ and 5′-AGTCTGACATCTCGACTC-3′ ) using 1X PCR buffer, 0.2 mM dNTPs and 1 U Taq DNA polymerase from Invitrogen (Karlsruhe, Germany) in a total volume of 25 µl and the following conditions: 4 min at 94°C, followed by 35 cycles of 30 sec at 94°C, 90 sec at 52°C, 90 sec at 72°C and a final extension of 30 min at 72°C. Genotypes were determined with an ABI 3100 Genetic Analyzer and GeneScan Genotyper version 3.7 NT software (Applied Biosystems, Darmstadt, Germany). Amplicons of detected alleles were cloned (TOPO TA Cloning Kit, Invitrogen) and sequenced (MWG Biotech, Ebersberg, Germany) to confirm sequence identity. The nucleotide sequences of two 437 bp fragments amplified with primers 5′- GAGTCAGTAACAGCTGCAG -3′ and 5′- AATCCAAGTGCAAGGCTG -3′ and representing different *Bos taurus* alleles were deposited in GenBank (AY752984 and AY752985). Additional sequencing with primers 5′-AGTCTAGAACAGCTGCAG-3′ and 5′-AATCCAAGTGCAAGGCT-3′ was performed to exclude microsatellite primer sequence polymorphism.

### RNA Isolation, Reverse Transcription and Imprinting Analysis

Tissue samples were homogenized in TRIZOL Reagent (Invitrogen) at 1 ml per 50 mg tissue following manufacturer’s instructions. The RNA was checked for integrity by agarose gel electrophoresis and quantified by A260 measurement. Twenty-five µg DNase I treated RNA (Trizol TM protocol, Invitrogen) were reverse transcribed in a 25 µL reaction with 400 U of SuperScript III enzyme (Invitrogen), 50 pmol of custom oligo-dT primer (5′-GAGATTTTTTTTTTTTTTTTTTTTVN-3′), 10 mM DTT (Invitrogen), 0.4 mM dNTPs and 20 U of RNase inhibitor (Promega, Mannheim, Germany). We used 0.25 µL cDNA in PCR reactions as described above to quantify expression of maternal and paternal alleles using the ABI 3100 Genetic Analyzer and GeneScan Genotyper software. We controlled for the possibility of genomic DNA contamination by performing PCR assays on RNA samples that had not been reverse transcribed and all results were negative (**Figure S3**).

Analysis of variance and *t*-tests were performed with SPSS 15. Differences were considered significant at *P*<0.05.

### DNA Methylation Analysis

Analysis of DNA methylation at 21 CpG sites in *IGF2R* DMR2 was performed using a combination of methylation-sensitive high resolution melting (MS-HRM) and pyrosequencing as described [37]. In brief, genomic DNA was bisulfite-converted using the EZ DNA Methylation-Gold Kit (Zymo Research, Irvine, CA, USA). The region of interest (**Figure 2A**) was amplified using the EpiTect HRM PCR Kit (Qiagen, Hilden, Germany) in a 25 µl reaction volume at 56°C annealing temperature. Primers were *in-silico* designed with the Pyromark Assay Design software version 2.0 (Qiagen) and tested for unbiased PCR-amplification utilizing artificially generated methylation standards [37]. Forward primer 5′-GGGGGTGGTTAAGGTTAGA-3′ and biotinylated reverse primer 5′-BIO-AACCTTCTCAACACCTTAC-3′ amplified a 287 bp segment of bovine *IGF2R* DMR2 [38]. The amplicon was purified using Wizard SV Gel and PCR Clean-Up System (Promega, Madison, WI, USA) and pyrosequenced on a Pyromark Q24 system (Qiagen) as described [39]. Sequencing primers for pyrosequencing were 5′-GTAGGAGTTAGAGTAGAAAT-3′ and 5′-GAAAGATAAGATATAATAGAG-3′.

## Supporting Information

Figure S1 **Nucleotide sequence alignment of human and cow** ***IGF2R*** **3′UTR from exon 48 with polymorphic microsatellite shaded.** Nucleotide positions for human (GenBank AF348209 positions 136669–137051, top row) and cow (GenBank AY752984 positions 1–437, bottom row) are indicated. Bars indicate nucleotide identity, dots denote indels and substitutions.
(TIF)

Figure S2 **Microsatellite fragment profiles obtained by loading increasing amounts (top to bottom) of the same PCR product.** This control experiment revealed low levels of expected contracted (TG)_n_ repeat (left arrow) but showed no evidence of expanded repeat (right arrow) that could have interfered with quantification of paternal *IGF2R* alleles. Fragment length scale in base pairs (bp) is indicated.
(TIF)

Figure S3 **Controls performed to exclude genomic DNA contamination of RNA samples.** Paired PCR assays of *IGF2R* 3′UTR microsatellite fragment with RNA samples that were reverse transcribed (cDNA) in comparison with samples (RNA) that had not been reverse transcribed. The gel at the top shows examples from all analyzed tissues; the gel at the bottom shows placenta samples that showed the highest expression from paternal alleles. Agarose gels were deliberately overloaded to visualize potentially small amounts of PCR product in non-reverse transcribed samples but all yielded negative results. M: Molecular size marker with fragment lengths in base-pairs (bp) indicated.
(TIF)
