# Supplementary figures and images for: Tissue-Specific and Minor Inter-Individual Variation in Imprinting of IGF2R Is a Common Feature of Bos taurus Concepti and Not Correlated with Fetal Weight
Source: PLoS One. 2013 Apr 8;8(4):e59564. doi: 10.1371/journal.pone.0059564 (PMC3620161; doi:10.1371/journal.pone.0059564)

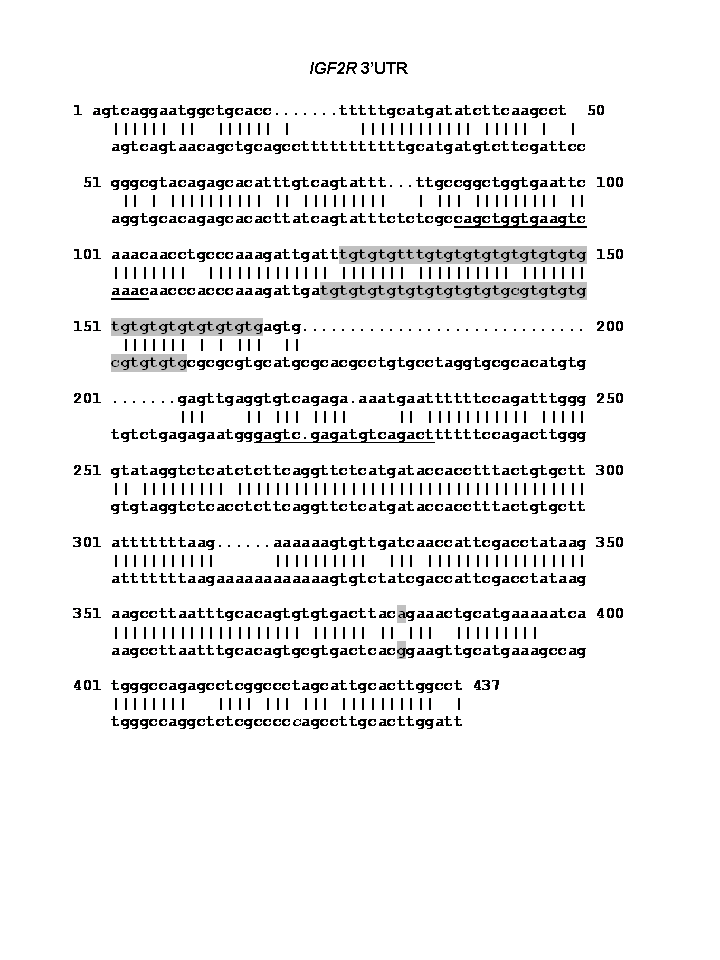

Supplement: Figure S1 — Nucleotide sequence alignment of human and cow IGF2R 3′UTR from exon 48 with polymorphic microsatellite shaded. Nucleotide positions for human (GenBank AF348209 positions 136669–137051, top row) and cow (GenBank AY752984 positions 1–437, bottom row) are indicated. Bars indicate nucleotide identity, dots denote indels and substitutions. (TIF) [file pone.0059564.s001.tif]
